# An update on factors affecting umbilical cord care among mothers: A review

**DOI:** 10.1097/MD.0000000000038945

**Published:** 2024-07-12

**Authors:** Emmanuel Ifeanyi Obeagu, Getrude Uzoma Obeagu

**Affiliations:** a Department of Medical Laboratory Science, Kampala International University, Ishaka, Uganda; b School of Nursing Science, Kampala International University, Ishaka, Uganda.

**Keywords:** babies, care, infection, mothers, neonatal sepsis, practices, umbilical cord

## Abstract

Umbilical cord care remains a critical aspect of newborn health, yet practices vary significantly across different cultures and healthcare settings. This paper aims to provide an updated synthesis of the factors influencing umbilical cord care among mothers. The umbilical cord is a vital link between the fetus and the placenta during pregnancy, but after birth, it requires proper care to prevent infections. Numerous factors influence a mother’s approach to umbilical cord care, including cultural beliefs, socio-economic status, access to healthcare information, and traditional practices passed down through generations. Understanding these factors is crucial for healthcare providers to offer tailored guidance and support to mothers, ensuring the optimal care for newborns. This paper examines recent research and literature encompassing diverse cultural perspectives, socio-economic considerations, healthcare access, and educational interventions related to umbilical cord care. It also highlights the impact of technological advancements, such as telemedicine and digital health platforms, in disseminating crucial information to mothers, especially in remote or underserved areas. Moreover, the review delves into the role of healthcare professionals in promoting evidence-based practices and addressing misconceptions regarding umbilical cord care. It emphasizes the importance of culturally sensitive and context-specific interventions in enhancing maternal knowledge and practices related to neonatal care. In conclusion, this review presents an updated overview of the multifactorial influences on umbilical cord care among mothers. It calls for continued research and concerted efforts to bridge gaps in knowledge, cultural beliefs, and healthcare access, ultimately contributing to the promotion of optimal newborn health outcomes.

## 1. Introduction

The umbilical cord is a special type of tissue that links the mother and unborn child through the placenta to exchange nutrients, carbon dioxide, and other metabolites.^[1–3]^ This cord needs to be cut, then dressed with sterile materials.^[4]^ According to published research, devitalized cord tissue can be an ideal environment for bacterial growth, particularly if it is kept moist and exposed to unclean materials like cow dung, roots from nearby trees, herb preparations, sand from doorposts, and herb preparations.^[5]^

Neonatal sepsis, which is caused by subpar cord care and kills 2 to 9 million newborns worldwide every year, has emerged as the main obstacle to further decreases in neonatal mortality.^[6]^ Thirteen percent of neonatal deaths worldwide are due to severe cord sepsis, which is one of the top 3 causes.^[7]^ In sub-Saharan Africa, it has been estimated that approximately 3.3 million neonatal deaths occur each year, of which more than 30% are brought on by infections, some of which begin as umbilical cord infections.^[8]^

Unhealthy traditional cord-care practices are frequently cited as a significant public health concern, and a study conducted in Zambia found that optimal umbilical cord care practices for newborns during the first week of life, especially in environments with poor hygiene, has the potential to prevent these preventable neonatal deaths.^[8]^ In areas and nations where nearly half of births take place at home, such as Uganda, this percentage is higher. Infections of the umbilical cord stump (omphalitis) are a significant cause of these infections in newly born babies in developing countries.^[9]^ In Uganda, more than 50% of mothers apply different substances to their babies’ umbilical cords to hasten the healing process, and the majority of mothers do not bathe their newborns for the first 24 hours.^[10]^

## 2. Social and demographic factors that influence umbilical cord care

Age, educational level, sociocultural orientation, and environmental factors are basic conditioning factors that can help or hinder a mother’s ability to provide care that is therapeutic. For example, older mothers may be more aware of clean cord care and have more experience with it.

### 2.1. Mothers’ ages

In one of the Nigerian studies, it was discovered that older mothers had good overall knowledge and practice of cord care.^[11]^ Mothers 35 years of age and older showed clean cord care, whereas babies whose mothers were 20 years of age or younger had higher rates of cord infections. Therefore, they came to the conclusion that older mothers were more likely to practice safe cord care, which led to better neonatal outcomes.^[12]^

### 2.2. Education

Students from the University of Nairobi conducted a related study at the Punwani Maternity Hospital in Kenya, which revealed that the mothers’ level of education and cultural attachment had a direct impact on how the neonates cared for their umbilical cords. The findings indicated that during the first 5 days after birth, the majority of mothers with low levels of education had their babies develop pus and redness on the umbilical cord.^[13]^ The level of education of mothers and clean cord management were found to be strongly correlated in another study. Higher educated mothers had more knowledge and used better cord care techniques. This is likely due to the fact that these mothers have access to some medical information from the media and other sources of information about child care, whereas uneducated women lack the ability to make their own decisions and are therefore unable to resist peer and family pressure, leading them to adhere to established tradition.^[14]^ The ability of mothers to make decisions that can encourage hygienic cord care and a reduction in umbilical cord infections increases with increased educational attainment.^[15]^

The level of education among mothers can significantly influence their understanding and adherence to clean cord management practices for newborns. Research has shown that higher levels of education often correlate with better health-seeking behaviors, improved hygiene practices, and increased access to healthcare information. Mothers with higher education levels often have better access to healthcare information and are more likely to be aware of recommended practices for umbilical cord care. They may understand the importance of cleanliness and hygiene in preventing infections and complications related to the umbilical cord stump. Educated mothers tend to follow healthcare provider recommendations more closely. They are more likely to comprehend instructions provided by healthcare professionals regarding proper cord care, including the importance of keeping the cord stump clean and dry. Higher education levels often correlate with better access to healthcare facilities, enabling mothers to seek professional advice or medical care promptly if they notice any abnormalities or signs of infection in the cord stump. Educated mothers may be more informed about the potential risks associated with improper cord care practices, such as infection transmission and delayed healing. This awareness may motivate them to adhere more diligently to recommended hygiene practices. However, it is essential to note that education level alone might not always determine a mother’s ability to provide optimal cord care. Other factors such as cultural beliefs, socioeconomic status, access to resources, and familial influences can also play significant roles in shaping caregiving practices. Healthcare providers play a crucial role in educating mothers from diverse educational backgrounds about proper cord care practices. Tailoring guidance to suit the individual circumstances, providing clear instructions, and addressing any misconceptions are vital aspects of ensuring optimal cord care, regardless of the mother’s education level.^[15]^

### 2.3. Practices by mothers on umbilical cord care

The practices and beliefs surrounding umbilical cord care vary, according to numerous studies. At the time of the study, only a small portion of sub-Saharan Africa population used dry cord care, as advised by the World Health Organization. This can be attributed to the region’s diverse cultures, each of which has a different perspective on how to care for newborns’ umbilical cords.^[16]^

There have been reports of a variety of substances being applied to cords; the reasons for these applications ranged from lubricating agents to drying agents to, finally, medicinal agents if an infection was suspected.^[16]^ A substance that would increase moisture or the “softness” of the cord may be taken into consideration if a cord stump is deemed to be excessively brittle, is cracking, or is bleeding. Breast milk, vaseline, cooking and motor oil, mabono (wild fruit) oil, and sour milk cream are a few examples.

Warm cooking oil or used motor oil were uncommon practices, but they were occasionally used as a base for other ingredients (e.g., vaseline), which was one of the most frequently applied products and frequently recommended by traditional birth attendants or grandmothers.

According to a study conducted in Nepal, the risk of infection was higher in infants who received topical mustard oil cord applications and other potentially unclean substances by 29 and 62 percent, respectively.^[17]^

Poor cord care is directly related to giving birth on a homestead because most of the time, these mothers are assisted by less qualified and less experienced people, such as traditional birth attendants. These frequently use local medicine that may not support proper cord care healing, predisposing this neonate to tetanus and ultimately causing death.^[17]^

According to a qualitative study conducted in Ethiopia, local cord care practices, particularly the concept of cord tying, which is used to stop blood flow and facilitate placenta delivery, are the main cause of neonatal mortality in the nation. On the cord, substances were applied to lubricate it, aid in its separation, and encourage healing. Delay in healing, bleeding, or swelling were recognized cord issues locally.^[18]^

Additionally, in southern Zambia, the cord of a healthy baby is treated with petroleum jelly, Manobo (wild fruit) oil, cooking/motor oil, charcoal, dried cow dung or chicken droppings, burned pumpkin stems, and crushed loma (wasps’ nest). If the cord is red or pus appears, a different group of substances that are regarded as medicinal are used. Python oil, breast milk, alcohol, bananas, cow dung, Mukunku (tree bark), conventional herbs, and dirt from a pounding stick are some examples of substances that are regarded as medicinal.^[16]^

According to a Tanzanian study, the umbilical cord can be bound or wrapped with a strip of cloth to keep it from touching the groin.^[19]^

The umbilical cord is a special tissue that connects the mother to the unborn child through the placenta for the exchange of nutrients, oxygen, carbon dioxide, and other metabolites. Cut the cord, then cover it with sterile supplies. It was discovered that the devitalized tissue of the cord can be an excellent medium for bacterial growth, particularly if the cord is kept moist and unclean substances like sand from door posts, herb preparations, cow dung, roots, or tree bark are applied to it.^[20]^

### 2.4. How is the cord tied off

The umbilical cord is typically clamped and then cut shortly after childbirth. This process involves 2 steps: clamping the cord and then cutting it. A healthcare provider, usually a nurse or midwife, uses a sterile plastic clamp or a specialized device to secure 2 points along the umbilical cord. This is usually done a few inches away from the baby’s belly, and another clamp may be applied a few inches further towards the placenta. This clamping process prevents bleeding from the umbilical vessels and allows for a safe separation of the cord. Once the cord is clamped, the healthcare provider uses sterile scissors or a cutting device to sever the cord between the 2 clamps. This action leaves a short length of the cord attached to the baby’s belly, which eventually dries up and falls off over the course of about one to 3 weeks.^[21]^

Regarding the term “dressed with sterile material,” after the cord is cut and clamped, the remaining part attached to the baby’s belly is usually left exposed to air to promote drying and healing. However, in some cases, especially if there’s a risk of infection or if the environment is not hygienic, the stump may be covered or “dressed” with a sterile material. “Dressing” the umbilical cord stump with sterile material involves placing a clean, sterile gauze pad or surgical dressing around the base of the cord where it attaches to the baby’s belly. This covering is intended to provide a barrier against external contaminants and reduce the risk of infection. The dressing is changed as needed to maintain cleanliness and prevent moisture buildup around the stump. It is important to note that healthcare practices related to umbilical cord care might slightly vary based on cultural traditions, healthcare facility protocols, or specific medical recommendations. Always follow the guidance provided by healthcare professionals to ensure proper care for the umbilical cord stump.^[22]^

### 2.5. Dry cord care

Dry cord care refers to a method of caring for the umbilical cord stump in newborns that involves keeping the area clean and allowing it to naturally dry and separate without applying any substances or materials. Keep the area around the umbilical cord stump clean. If necessary, gently clean the area with water and mild soap. Pat it dry gently with a clean, soft cloth or gauze. Allow the cord stump to remain exposed to air as much as possible. This exposure facilitates drying and helps the stump to naturally dry up and fall off.^[22]^ During bathing, it’s advisable to give the baby sponge baths rather than immersing them in water until the cord stump falls off. This prevents the stump from being soaked and aids in the drying process. Refrain from applying substances like alcohol, antiseptics, lotions, or powders unless specifically recommended by a healthcare professional. These substances can interfere with the natural drying process and may increase the risk of irritation or infection. Dry cord care is a widely practiced method that is often recommended by healthcare providers, as it aligns with the natural healing process of the umbilical cord stump. It minimizes the risk of infection and allows the cord stump to detach naturally, usually within one to 3 weeks after birth. However, it’s essential to monitor the cord stump regularly for any signs of infection, such as redness, swelling, discharge, or foul odor. If you notice any concerning changes or have any doubts, it is crucial to seek advice from a healthcare provider promptly. Always follow the specific guidance provided by your healthcare professional regarding cord care, as practices may vary based on individual circumstances or medical recommendations.^[22]^

### 2.6. Optimal cord care practices

Optimal cord care practices aim to promote the healing and prevent infection of the umbilical cord stump in newborns.^[23]^ Here are some key practices recommended by healthcare professionals:

*Hand hygiene*: Before handling the baby’s umbilical cord stump, always wash your hands thoroughly with soap and water or use hand sanitizer to prevent introducing harmful bacteria.

*Keep it clean and dry*: Keep the area around the cord stump clean and dry. Ensure the diaper does not cover the stump to allow air circulation, which aids in drying and healing. Clean the area gently with water if necessary, and avoid using alcohol, antiseptics, or powders unless advised by a healthcare professional.

*Proper handling*: Handle the baby gently to prevent unnecessary tugging or pulling on the cord stump. Avoid dressing the baby in clothes that rub against or irritate the cord area.

*Frequent checking*: Monitor the cord stump regularly for any signs of infection. Signs of infection may include redness, swelling, foul odor, or discharge. If you notice any concerning changes, contact a healthcare provider immediately.

*Cord care in bathing*: It is generally recommended to give sponge baths until the cord stump falls off to avoid submerging it in water, which might delay drying. Pat the area dry gently after the bath.

*Allow natural healing*: Let the cord stump fall off naturally. Avoid pulling or forcing it off, even if it seems loose or hanging.

*Follow healthcare provider’s advice*: Follow the specific instructions provided by your healthcare provider. They might have tailored advice depending on your baby’s health and individual circumstances.

*Promote general hygiene*: Ensure overall cleanliness and hygiene in the baby’s environment, including clean bedding, clothing, and a clean changing area, to reduce the risk of infection.

*Educate and involve caregivers*: Educate caregivers, family members, and anyone involved in the care of the newborn about proper cord care practices to ensure consistency in care.

Remember, while these practices are generally recommended, always consult your healthcare provider for specific guidance tailored to your baby’s needs. Additionally, guidelines might vary based on cultural practices or healthcare provider recommendations, so it is essential to follow the advice provided in your specific situation.

### 2.7. Recommendations

Always wash hands thoroughly with soap and water or use hand sanitizer before handling the baby’s umbilical cord stump. Keep the area around the cord stump clean and dry. Ensure that the diaper does not cover the stump to allow air circulation, aiding in drying and healing. Handle the baby gently to prevent tugging or pulling on the cord stump. Avoid dressing the baby in clothes that rub against or irritate the cord area. Give sponge baths instead of submerging the baby in water until the cord stump falls off to prevent moisture accumulation. Allow the cord stump to fall off naturally. Avoid pulling or forcing it off, even if it seems loose or hanging. Regularly check the cord stump for signs of infection such as redness, swelling, discharge, or foul odor. Contact a healthcare provider if you notice any concerning changes. Refrain from applying alcohol, antiseptics, lotions, or powders unless specifically advised by a healthcare professional. Adhere to the specific instructions provided by your healthcare provider for caring for the umbilical cord stump. Educate family members and caregivers about proper cord care practices to ensure consistency in care. Maintain cleanliness in the baby’s environment, including clean bedding, clothing, and a clean changing area, to reduce the risk of infection. Remember, while these recommendations are generally accepted, it’s crucial to follow the advice provided by healthcare professionals as guidelines may vary based on individual circumstances or medical recommendations. Always seek guidance if you have any concerns or questions about caring for your baby’s umbilical cord stump.

## 3. Conclusion

The diverse array of factors influencing umbilical cord care practices among mothers highlights the complexity of this essential aspect of neonatal health. Cultural beliefs, socio-economic disparities, healthcare access, and the intergenerational transfer of traditions significantly impact how mothers approach caring for their newborns’ umbilical cords. Recognizing these multifaceted influences is crucial for healthcare providers, policymakers, and stakeholders to design effective interventions. Tailored educational programs that consider cultural contexts, enhance healthcare access, and promote evidence-based practices are imperative to improve maternal knowledge and adherence to safe cord care methods. Advancements in technology, including telemedicine and digital health platforms, present promising avenues for disseminating accurate information, particularly in remote or underserved areas. However, these initiatives should be implemented with cultural sensitivity and community involvement to ensure acceptance and effectiveness.

Healthcare professionals play a pivotal role in debunking misconceptions, providing accurate guidance, and encouraging mothers to adopt safe cord care practices. Collaborative efforts among healthcare providers, policymakers, community leaders, and mothers themselves are essential to bridge knowledge gaps and foster a shared understanding of optimal cord care practices. Moving forward, continued research efforts and a concerted focus on culturally sensitive interventions are needed to address disparities, promote positive behaviors, and ultimately improve newborn health outcomes worldwide. By acknowledging and addressing the diverse influences shaping umbilical cord care practices, we can strive towards ensuring the best possible start for every newborn’s health and well-being.

## Author contributions

**Conceptualization:** Emmanuel Ifeanyi Obeagu.

**Methodology:** Emmanuel Ifeanyi Obeagu, Getrude Uzoma Obeagu.

**Supervision:** Emmanuel Ifeanyi Obeagu.

**Validation:** Emmanuel Ifeanyi Obeagu.

**Visualization:** Emmanuel Ifeanyi Obeagu, Getrude Uzoma Obeagu.

**Writing – original draft:** Emmanuel Ifeanyi Obeagu, Getrude Uzoma Obeagu.

**Writing – review & editing:** Emmanuel Ifeanyi Obeagu, Getrude Uzoma Obeagu.
